# Differences in the Efficacy of Cognitive Function Treatment Related to Functional Patterns in the Frontal‐Limbic Network in Patients With Bipolar Disorder

**DOI:** 10.1002/cns.70730

**Published:** 2026-01-05

**Authors:** Sujuan Li, Yangpan Ou, Haiping Liu, Qianyu Dong, Yan Qiu, Ziwei Teng, Hui Tang, Hui Xiang, Guowei Wu, Jindong Chen, Bolun Wang, Lutao Jiang, Haishan Wu

**Affiliations:** ^1^ Department of Psychiatry, National Clinical Research Center for Mental Disorders, and National Center for Mental Disorders The Second Xiangya Hospital of Central South University Changsha Hunan China; ^2^ Qingdao Mental Health Center Qingdao Shandong China; ^3^ Xiamen Xianyue Hospital, Xianyue Hospital Affiliated With Xiamen Medical College, Fujian Psychiatric Center, Fujian Clinical Research Center for Mental Disorders Xiamen Fujian China; ^4^ Department of Psychiatry, Clinical Research Center for Depressive Disorder in Hunan Province The Second People's Hospital of Hunan Province (Brain Hospital of Hunan Province) Changsha China; ^5^ Department of Radiology The Second Xiangya Hospital of Central South University, Clinical Research Center for Medical Imaging in Hunan Province, Scientific Research Program of Hunan Provincial Health Commission Changsha Hunan China; ^6^ The Third People's Hospital of Tongren Tongren Guizhou China

**Keywords:** connectome‐based predictive modeling, degree centrality, regional homogeneity, repeatable battery for the assessment of neuropsychological status, support vector machine

## Abstract

**Background:**

This study aimed to identify neurobiomarkers that predict the efficacy of treatment of cognition in patients with bipolar disorder (BD).

**Methods:**

Regional homogeneity (ReHo) and degree centrality (DC) values, which are two functional magnetic resonance imaging indicators, were analyzed to compare differences in brain activities between patients with BD and healthy controls (HC).

**Results:**

BD patients (*N* = 92) exhibited increased activity in the right hippocampus or right parahippocampal gyrus compared to HC, while their ReHo and DC values in the left middle frontal gyrus decreased in the resting state. The delayed memory scores were predicted by using connectome‐based predictive modeling in patients with BD at baseline. After 12 weeks of treatment, the patients with BD, whose cognitive function improved (*n* = 24), showed activity in the right superior temporal gyrus (STG) and left anterior cingulate cortex (ACC) at baseline. The improvement in cognitive function of patients with BD is distinguished by abnormal activities using support vector machine analysis.

**Conclusions:**

The abnormal frontal‐limbic network plays a critical role in the underlying neuropathological mechanism of cognitive impairment in patients with BD. The right STG and left ACC hold the potential to serve as neurobiomarkers for predicting the clinical efficacy of cognitive function treatment. These regions provide additional target options for future physical treatments of cognitive impairment in patients with BD.

## Graphical abstract

Graphical Abstract of Differences in the Efficacy of Cognitive Function Treatment Related to Functional Patterns in the Frontal‐Limbic Network in Patients with Bipolar Disorder.

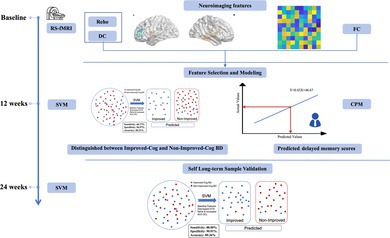

## 1 Introduction

Bipolar disorder (BD), also known as a chronic affective disorder, is characterized by recurrent episodes of depression and mania. The underlying mechanisms are not yet fully understood, and current treatment approaches need to be improved [1]. Apart from affective symptoms, cognitive impairment is commonly observed in patients with BD. This cognitive impairment contributes to functional disability and high rates of unemployment [2, 3, 4], reflecting mood disorders associated with the primary socioeconomic burdens [5, 6]. Studies have suggested that changes in brain structure and function may be attributed to the cognitive impairment observed in patients with BD [7]. However, there is a lack of effective treatment options that improve cognition.

One of the reasons for this limitation is the absence of valid and reliable neural circuit‐based biomarkers for pro‐cognitive effects [8]. Thus, the International Society for Bipolar Disorder organized an international task force to develop consensus‐based guidelines for the design and methodology of cognition trials. In their recommendations, neuroimaging assessments are adopted in future trials to explore treatment‐related target engagement within the neural circuitry underlying cognitive impairment in patients with BD [9]. In addition, a repetitive transcranial magnetic stimulation strategy was developed using objective biomarkers to obtain encouraging outcomes [10]. Therefore, it is crucial to find biomarkers that predict the treatment efficacy of cognitive function. It serves as intermediate endpoints in the treatment of cognitive impairment in patients with BD.

Regional homogeneity (ReHo) is a well‐established data‐driven method for assessing regional brain activities [11]. Degree centrality (DC) analysis is used to assess global connectivity of a brain region with the rest of the brain [12]. ReHo and DC methods provide indicators that reflect regional brain activity. Previous studies have identified abnormal regional activities in the hippocampus [13], left anterior cingulate cortex (ACC) [14], amygdala [15], and medial prefrontal cortex (mPFC) [16] in patients with BD. However, results given by these studies regarding different functional indicators in patients with BD are inconsistent. Some studies have reported decreased ReHo values in the right inferior temporal gyrus in patients with BD [17], while others have found increased fractional amplitude of low‐frequency fluctuation values in the same region [18]. Nevertheless, these research findings gave the same suggestion that abnormalities in the functional patterns of the frontal‐limbic network in patients with BD were detected. Prior studies have suggested that cognitive impairment in patients with BD is associated with abnormalities in various brain regions with regard to structure and function. The abnormal functioning of the frontal‐limbic network may be the cause of the cognitive impairments observed in these patients [19]. For example, abnormal brain functional connectivity (FC) was associated with memory recall in patients with depressive episodes following treatment [20]. Moreover, pediatric patients with BD have exhibited increased activation in the inferior frontal gyrus and decreased activation in limbic regions during attentional tasks [21].

In other studies, cognitive impairment in patients with BD associated with structural abnormalities in the brain was reported. For example, Suzanne et al. found that hippocampal volume was positively correlated with relational memory performance in patients with BD [7]. Furthermore, the thickness of the dorsal prefrontal cortex before treatment predicted the improvement of executive function [22]. These findings highlight the role of the frontal‐limbic system in the neurobiological mechanisms underlying cognitive impairment in patients with BD. The frontal lobe establishes connections with the limbic lobe. The PFC plays a vital role in generation and regulation of emotions, particularly those from the limbic system. The limbic system is involved in motivation, emotion, learning, and memory [23]. The limbic system includes the amygdala, hippocampus, thalamus, cingulate gyrus, and nucleus accumbens, linking the subcortical structures and the cerebral cortex [23, 24]. In particular, the ACC has received particular attention, for it holds significant importance in mood disorders [25]. A study found that increased FC between the right medial orbitofrontal cortex and the left ACC was correlated with a positive response to lithium monotherapy in patients with BD [26]. The hippocampus plays vital roles in memory, cognition, and stress regulation. Another study demonstrated that increased FC in the right hippocampus after electroconvulsive therapy was associated with clinical improvement in patients with varying levels of unipolar depression [27]. Overall, tasks related to emotion processing, reward processing, and working memory rely on the activation of frontal‐limbic brain regions [28, 29, 30, 31].

Connectome‐based predictive modeling (CPM) is a data‐driven approach that links brain connectivity with behavior for developing predictive models [32]. In several studies, the CPM protocol was applied to show robust relationships between brain connectivity, fluid intelligence [33], and sustained attention [34]. It is demonstrated that its establishment of relationships between brain and behavior/cognitive function through cross‐validation is effective. Using the CPM protocol, one study predicted the severity of depressed and elevated mood in patients with BD based on distributed functional connectomes [35]. A support vector machine (SVM) is a supervised learning model that utilizes learning algorithms to analyze data for regression and classification [36]. SVM is used for regression, classification, and outlier detection. It has been widely used in the classification and prediction of psychiatric diseases. For example, Wang et al. achieved high classification accuracy in identifying individuals with BD from healthy controls (HC) using a valid radiomics approach with SVM [37]. By combining structural and functional MRI data, SVM classifiers can identify patients with BD in clinical settings [38].

Previous studies focused on improving emotional symptoms in patients with BD and paid little attention to the effect of cognitive function on their social functioning. Moreover, predictive indicators for the treatment efficacy of cognitive function are lacking. In this connection, this study aims to investigate the relationship between cognitive function by comparing the differences in brain function between drug‐naive patients with BD and HC. Furthermore, CPM was used to establish a model for predicting cognitive function scores in patients with BD. This study aimed to develop biomarkers that predict the treatment efficacy of cognitive function in patients with BD.

## 2 Methods

**FIGURE 1:**
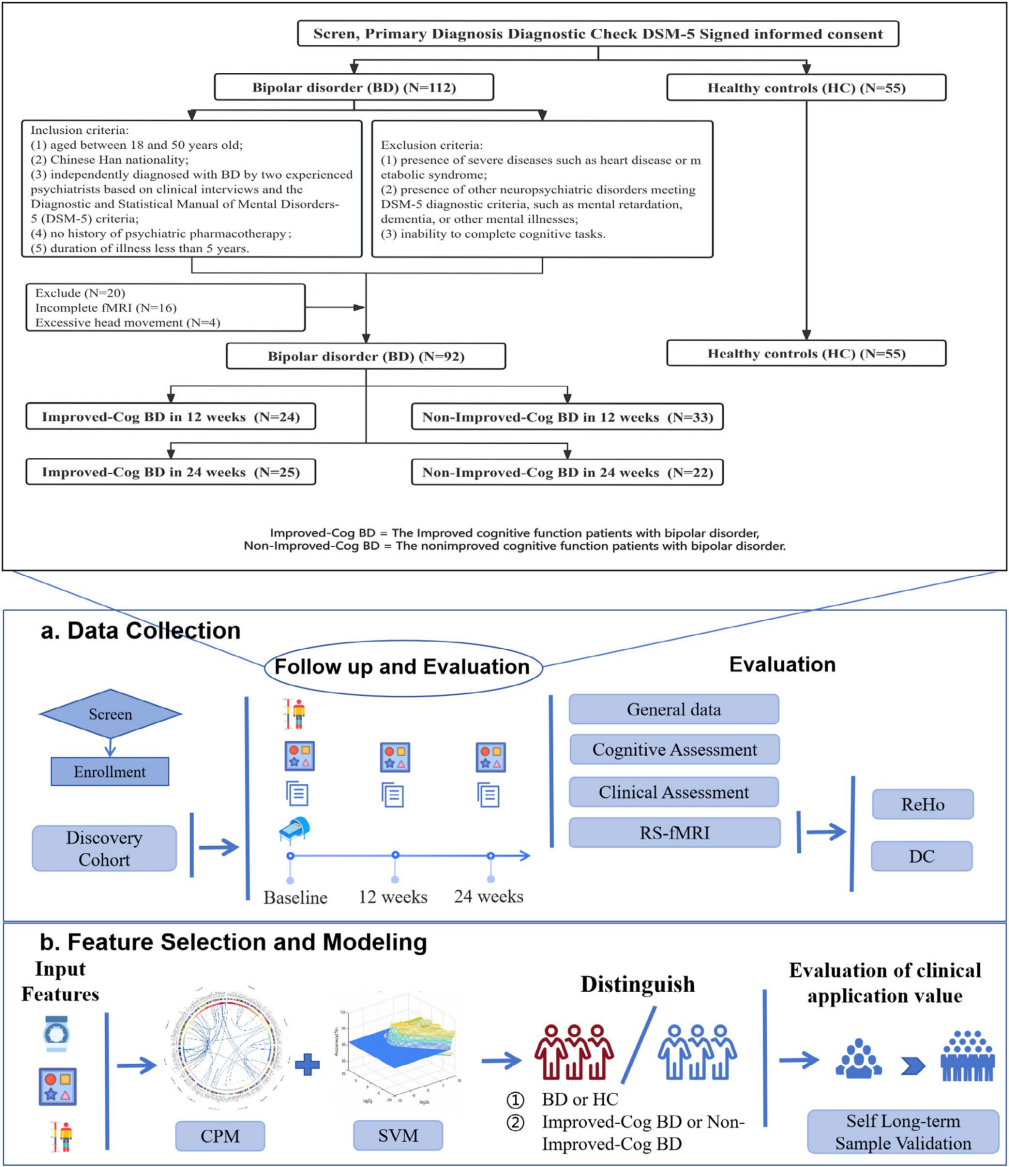
Flow chart. BD, the patients with bipolar disorder; CPM, connectome‐based predictive modeling; DC, degree centrality; HC, healthy controls; ReHo, regional homogeneity; SVM, support vector machine.

The overall study design is presented in Figure 1. Detailed methods are described as follows:

### 2.1 Participants

This study recruited drug‐naive patients with BD who were initially diagnosed at the Second Xiangya Hospital of Central South University from March 2019 to December 2022. The inclusion criteria for patients were as follows: (1) aged between 18 and 50 years old, (2) of Chinese Han nationality, (3) independently diagnosed with BD by two experienced psychiatrists based on clinical interviews and the Diagnostic and Statistical Manual of Mental Disorders‐5 (DSM‐5) criteria, (4) no history of psychiatric pharmacotherapy, and (5) duration of illness less than 5 years. The exclusion criteria were as follows: (1) presence of severe diseases such as heart disease or metabolic syndrome; (2) presence of other neuropsychiatric disorders meeting the DSM‐5 diagnostic criteria, such as mental retardation, dementia, or other mental illnesses; and (3) inability to complete cognitive tasks.

Sex‐, age‐, and education‐matched HC were recruited from the local community. Detailed information on the inclusion criteria for HC is shown in Appendix S1, Methods 2.1.

### 2.2 Clinical and Cognitive Assessment

Symptoms related to BD were assessed using the Hamilton Depression Scale‐17 [39], Young Mania Rating Scale [40], and Hypomania Check List [41, 42]. Cognitive function was evaluated using the Repeatable Battery for the Assessment of Neuropsychological Status (RBANS) and Stroop color‐word test (Stroop). None of the participants received physical treatments such as Electroconvulsive Therapy (ECT) during the study period. Detailed information of the aforementioned assessment tools is shown in Appendix S1, Methods 2.2. Clinical and cognitive function assessments were conducted at baseline, 12 weeks, and 24 weeks during a 24‐week follow‐up period.

### 2.3 Assessing Clinical Improvement in Cognitive Function

To assess whether patients' cognitive function had reached the standard of clinically significant improvement at 12 and 24 weeks, two criteria were used: the reliable change index and a cutoff value. Researchers have also used these criteria to assess cognitive function improvement [43, 44].

The reliable change index quantifies the observed difference in a scale between baseline and follow‐up that exceeds the amount of change caused by measurement error [43]. The calculation method is as follows:

$${\mathrm{RC}}_{\text{index}}=1.96\times {\mathrm{SE}}_{\text{diff}},\;{\mathrm{SE}}_{\text{diff}}={\mathrm{SD}}_{1}\times \sqrt{2}\times \sqrt{1-\alpha }$$

SD_1_ is the standard deviation of the RBANS at baseline; Cronbach's α is the reliability of the RBANS.

A cutoff point for clinically significant change was determined by applying a formula that equates the probability of belonging to either the dysfunctional or functional population. It is written as follows:

$${\mathrm{CS}}_{\mathrm{cut}-\mathrm{off}}=\frac{\left({\text{Mean}}_{\text{clin}}\times {\mathrm{SD}}_{\mathrm{norm}}\right)+\left({\text{Mean}}_{\mathrm{norm}}\times {\mathrm{SD}}_{\text{clin}}\right)}{{\mathrm{SD}}_{\mathrm{norm}}+{\mathrm{SD}}_{\text{clin}}},$$

where “clin” and “norm” stand for patients with BD and the HC, respectively.

In this study, an improvement in cognitive function was defined by meeting the following criteria: (1) a difference in RBANS scores compared with baseline at 12 and 24 weeks, (2) RBANS change scores exceeding the reliable change index, and (3) RBANS scores at 12 and 24 weeks surpassing the cutoff point. Based on the abovementioned criteria, patients with BD were categorized into two groups: the improved cognitive function patients with BD (Improved‐Cog BD) group and the nonimproved cognitive function patients with BD (Non‐Improved‐Cog BD) group.

### 2.4 Image Acquisition and Imaging Data Processing

MRI images were acquired using a 3T MRI scanner (Siemens Verio, Erlangen, Germany) while participants remained motionless and awake with their eyes closed, aided by soft earplugs and foam pads to reduce scanner noise and head motion. Details of data acquisition and preprocessing can be found in Appendix S1, Methods 2.4.

### 2.5 ReHo and DC Analysis

The DPARSF toolbox was used to conduct ReHo analysis [45]. In this analysis, individual ReHo maps was generated by synchronizing the time series of each voxel with its nearest 26 neighbors based on the Kendall's coefficient of concordance (KCC) method. To account for individual variations, the ReHo maps were normalized by dividing the KCC for each voxel by the average KCC of the whole brain for each subject when individual variations were taken into account. Then, the ReHo maps were smoothed with a 4 mm full width at half maximum Gaussian kernel to reduce noise and residual differences.

For DC analysis, voxel‐based whole‐brain Pearson correlation was performed by analyzing the preprocessed images with a correlation coefficient threshold of *r* > 0.25, employing the Data Processing Assistant for Resting‐State fMRI (DPARSF) toolbox. Only positive correlation coefficients were considered in the study. The DC values for each voxel were obtained by summing the significant correlations. Subsequently, the DC values were subjected to Fisher‐Z transformation to enhance their normality. The calculation of DC followed the method described in a previous study by Zuo et al. [46].

### 2.6 CPM Analysis

The functional magnetic resonance imaging (fMRI) data recorded were divided into 246 anatomical regions of interest using the human Brainnetome Atlas (BNA). This atlas provided a fine‐grained, cross‐validated atlas that separates the brain into 210 cortical and 36 subcortical subregions by introducing anatomical and FC information [47]. For each subject, a representative time series in each region was obtained by averaging the fMRI time series of all voxels in each of the 246 regions [48]. FC between each pair of regions was evaluated using Pearson's correlation coefficients, resulting in (246 × 246) connectivity matrices for each subject. CPM was performed using published MATLAB scripts to predict the scores of the RBANS [32]. Detailed steps of the CPM analysis are given in Appendix S1, Methods 2.6.

### 2.7 Clinical Correlation

Partial correlation analysis was conducted to examine the correlation between abnormal ReHo and DC values and clinical variables at baseline in both patients with BD and HC. Age, gender, education level, and framewise displacement (FD) value were included as covariates. Bonferroni correction was applied to the correlation analyses in both groups.

### 2.8 SVM Analysis

SVM analysis was used to determine abnormal ReHo and DC values at baseline that distinguished between the Improved‐Cog BD and Non‐Improved‐Cog BD groups after 12 weeks and 24 weeks of treatment. In SVM analysis, a training dataset was used to distinguish between patients and controls and evaluates the classification performance on new, unseen data using a testing dataset. The classifier algorithm was applied with a leave‐pair‐out cross‐validation method to optimize specificity and sensitivity [49]. SVM [50] analysis was conducted to assess the classification accuracy of abnormal ReHo and DC values.

### 2.9 Statistical Analysis

Demographic and clinical data were analyzed using SPSS 24.0, with statistical significance set at *p* < 0.05. Homogeneity and normality of variance were tested for the variables. Two‐sample *t*‐tests (for continuous variables) and chi‐square tests (for categorical variables) were used to compare demographic and clinical information and cognitive function among groups. One‐way analysis of variance (ANOVA) was used to compare differences in age, gender, education level, clinical variables, RBANS total score, and dimension scores at baseline among the Improved‐Cog BD, Non‐Improved‐Cog BD, and HC groups.

For the imaging data, the DPARSF toolbox was used for statistical analysis. Two‐sample *t*‐tests were used to compare ReHo and DC values between patients with BD and HC. One‐way ANOVA and post hoc *t*‐tests were conducted to identify intergroup differences in ReHo values and DC maps among the three groups. Mean FD, age, gender, and educational years were included as covariates. The significance level (*p* value) was corrected for multiple comparisons based on the Gaussian random field theory (voxel significance: *p* < 0.001, cluster significance: *p* < 0.05).

## 3 Results

### 3.1 Demographic and Clinical Characteristics

A total of 112 patients with BD and 55 HC were initially recruited for this study. However, 16 patients with BD did not complete fMRI scans, and four patients with BD were excluded because of excessive head movements, resulting in a final sample size of 92 patients with BD and 55 HC. Of the participants with BD, 36 (39.1%) had bipolar I disorder, and 56 (60.9%) had bipolar II disorder. The majority of patients (68.5%) were in a depressed episode, followed by manic or hypomanic episodes (7.6%) and mixed or subthreshold mixed episodes (23.9%). No significant differences in demographic characteristics such as gender composition, age, and education years were found among the three groups (Table S1). In addition, no significant differences in gender, age, education level, and FD were found between patients with BD and HC. Furthermore, patients with BD exhibited lower scores on all RBANS measures and Stroop measures, except for line orientation, picture naming, coding tasks, and list recognition (Table 1). No differences in RBANS total score, RBANS T value, and Stroop total score were found among different BD disease phases (Table S1).

**TABLE 1:** Demographic and clinical characteristics data at baseline.^a^

Variable	HC (*n* = 55)	BD (*n* = 92)	*t* or χ^2^ ^b^	*p*
Demographic characteristic				
Gender (M, W)	(20, 35)	(25, 67)	1.369	0.242
Age (y)	20.60 ± 1.62	20.55 ± 3.96	0.098	0.992
Education(y)	14.32 ± 1.04	13.89 ± 2.06	1.703	0.091
Disease duration(y)	—	2.90 ± 1.52	—	—
Framewise displacement	0.07 ± 0.02	0.08 ± 0.03	−1.970	0.051
Clinical characteristic				
HAMD‐17	—	22.66 ± 7.58	—	—
YMRS	—	10.09 ± 6.84	—	—
HCL‐32	—	19.01 ± 6.31	—	—
RBANS T Value	94.38 ± 9.27	84.55 ± 10.32	5.801	< 0.001
RBANS Total Score	481.75 ± 34.73	442.16 ± 42.21	5.886	< 0.001
Immediate Memory	98.04 ± 13.90	83.82 ± 14.01	5.974	< 0.001
List Learning	32.56 ± 3.78	28.78 ± 5.17	4.475	< 0.001
Story Memory	17.55 ± 3.17	13.88 ± 3.92	5.877	< 0.001
Visuospatial	75.06 ± 8.92	70.74 ± 7.77	3.081	0.002
Figure Copy	16.02 ± 1.13	14.52 ± 1.44	6.583	< 0.001
Line Orientation	16.42 ± 2.92	15.99 ± 2.93	0.860	0.391
Language	95.76 ± 12.80	88.14 ± 16.09	2.992	0.003
Picture Naming	9.09 ± 0.80	8.92 ± 0.88	1.151	0.252
Semantic Fluency	21.47 ± 4.30	19.49 ± 4.46	2.643	0.009
Attention	117.89 ± 11.40	111.86 ± 12.32	2.945	0.004
Digit Span	61.98 ± 9.06	57.36 ± 9.30	2.945	0.004
Coding Tasks	15.22 ± 1.10	15.01 ± 1.30	0.991	0.323
Delayed Memory	95.00 ± 6.03	87.61 ± 10.52	5.412	< 0.001
List Recall	8.11 ± 1.74	7.15 ± 1.89	3.063	0.003
List Recognition	19.84 ± 0.42	19.70 ± 0.81	1.386	0.168
Story Recall	9.69 ± 1.75	7.89 ± 2.20	5.170	< 0.001
Figure Recall	14.31 ± 1.55	12.45 ± 2.86	5.112	< 0.001
Stroop Total Score	229.13 ± 30.50	210.39 ± 34.36	3.315	0.001
Word‐reading	105.40 ± 13.16	97.58 ± 17.07	3.084	0.002
Color‐naming	77.16 ± 13.61	71.47 ± 13.10	2.496	0.014
Color‐word	46.56 ± 10.82	41.34 ± 10.10	2.936	0.005

Abbreviations: BD, the patients with bipolar disorder; HC, healthy controls.

^a^ Data are presented as mean ± SD.

^b^ Test statistics: χ^2^ test for categorical variables and Student t‐test for continuous variables.

### 3.2 Differences in ReHo and DC Between BD and HC

**FIGURE 2:**
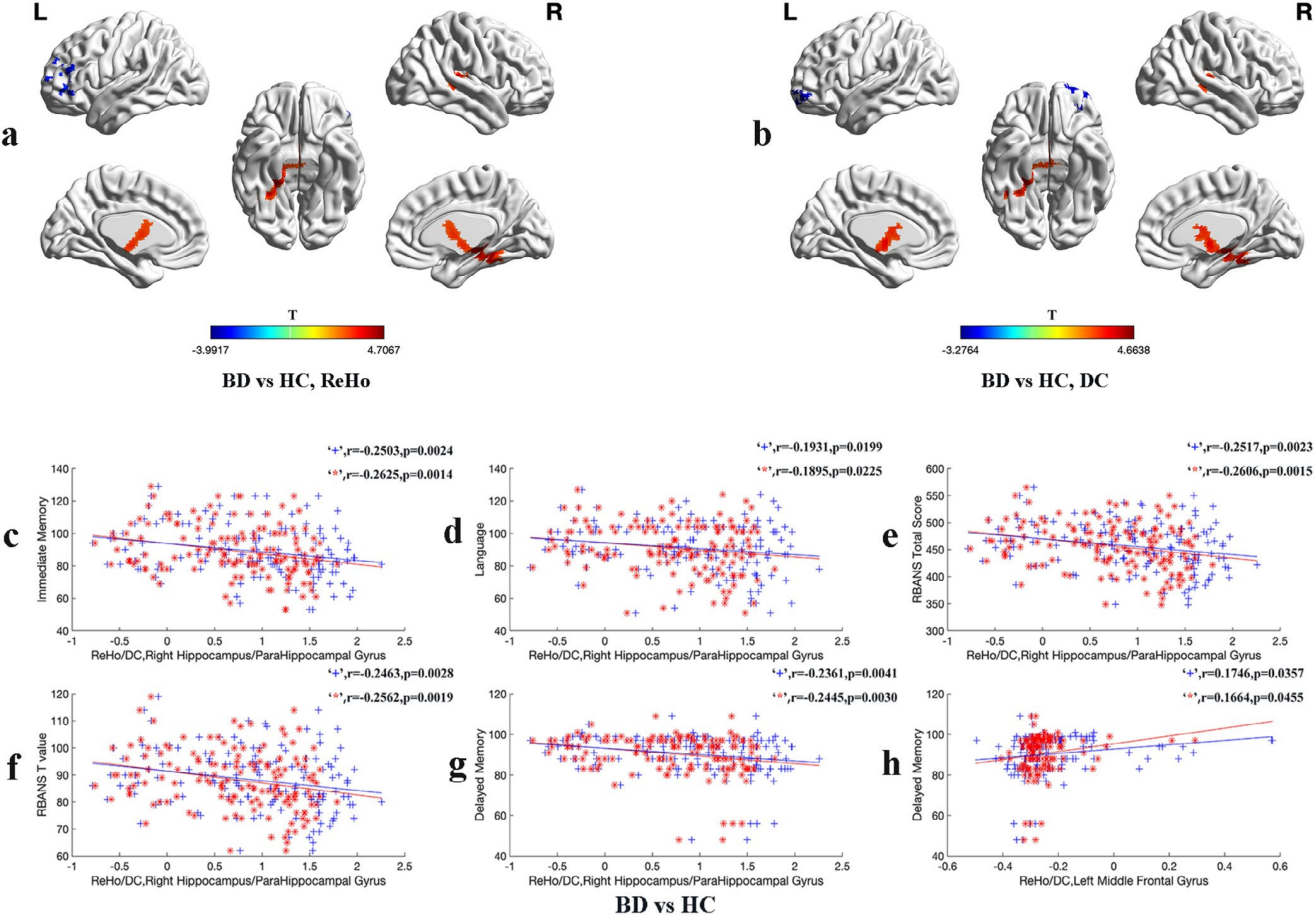
ReHo and DC Alterations in BD versus HC: Group Differences and Clinical Associations. (a, b) Brain regions showing significant ReHo (left) and DC (right) differences between patients with BD and HC. Red and blue denote higher and lower ReHo/DC values respectively in patients with BD compared to the HC. The color bars indicate the T values of the analysis of two‐sample *t*‐test. (c–h) Pearson correlation analysis was used to calculate the correlation between abnormal ReHo and DC values and clinical variables in patients with BD and the HCs. Age, gender, education level, and Framewise Displacement (FD) value were used as covariates. In both cases, the correlation analyses were Bonferroni corrected. “+” denotes ReHo values and “*” denotes DC values. BD, the patients with bipolar disorder; DC, degree centrality; HC, healthy controls; ReHo, regional homogeneity.

The results revealed that patients with BD showed increased ReHo and DC values in the right hippocampus/right parahippocampal gyrus compared with HC. Conversely, patients with BD exhibited decreased ReHo (Figure 2a) and DC values (Figure 2b) in the left middle frontal gyrus (Table 2).

**TABLE 2:** Significant Differences in ReHo and DC across Groups.

Measure, and Cluster location	Peak (MNI)			Number of voxels	*T* value
	x	y	z		
ReHo					
BD versus HC					
Right Hippocampus/Right ParaHippocampal Gyrus	33	−30	0	124	4.7607
Left Middle Frontal Gyrus	−51	42	18	84	−3.9917
Improved‐Cog BD versus HC					
Right Hippocampus/Right ParaHippocampal Gyrus	15	−24	−12	37	3.1420
Left Putamen/Left Thalamus	−15	−9	0	71	3.5472
Non‐Improved‐Cog BD versus HC					
Right Hippocampus/Right ParaHippocampal Gyrus	24	−36	−18	41	4.1362
Improved‐Cog BD versus Non‐Improved‐Cog BD					
Right Superior Temporal Gyrus	78	−9	6	30	−3.7002
DC					
BD versus HC					
Right Hippocampus/Right ParaHippocampal Gyrus	33	−30	−3	126	4.6638
Left Middle Frontal Gyrus, Orbital Part	−30	45	−12	82	−3.2764
Improved‐Cog BD versus HC					
Right Hippocampus/Right ParaHippocampal Gyrus	45	−39	−3	26	3.1408
Left Putamen/Left Thalamu	−15	−9	0	71	3.9122
Non‐Improved‐Cog BD versus HC					
Right Hippocampus/Right ParaHippocampal Gyrus	24	−36	−21	62	4.6351
Improved‐Cog BD versus Non‐Improved‐Cog BD					
Left Anterior Cingulate Gyrus	−24	33	3	14	3.7552

Abbreviations: DC, degree centrality; HC, healthy controls; Improved‐Cog BD, the improved cognitive function patients with bipolar disorder; MNI, Montreal Neurological Institute; Non‐Improved‐Cog BD, the nonimproved cognitive function patients with bipolar disorder; ReHo, regional homogeneity.

### 3.3 CPM Analysis

**FIGURE 3:**
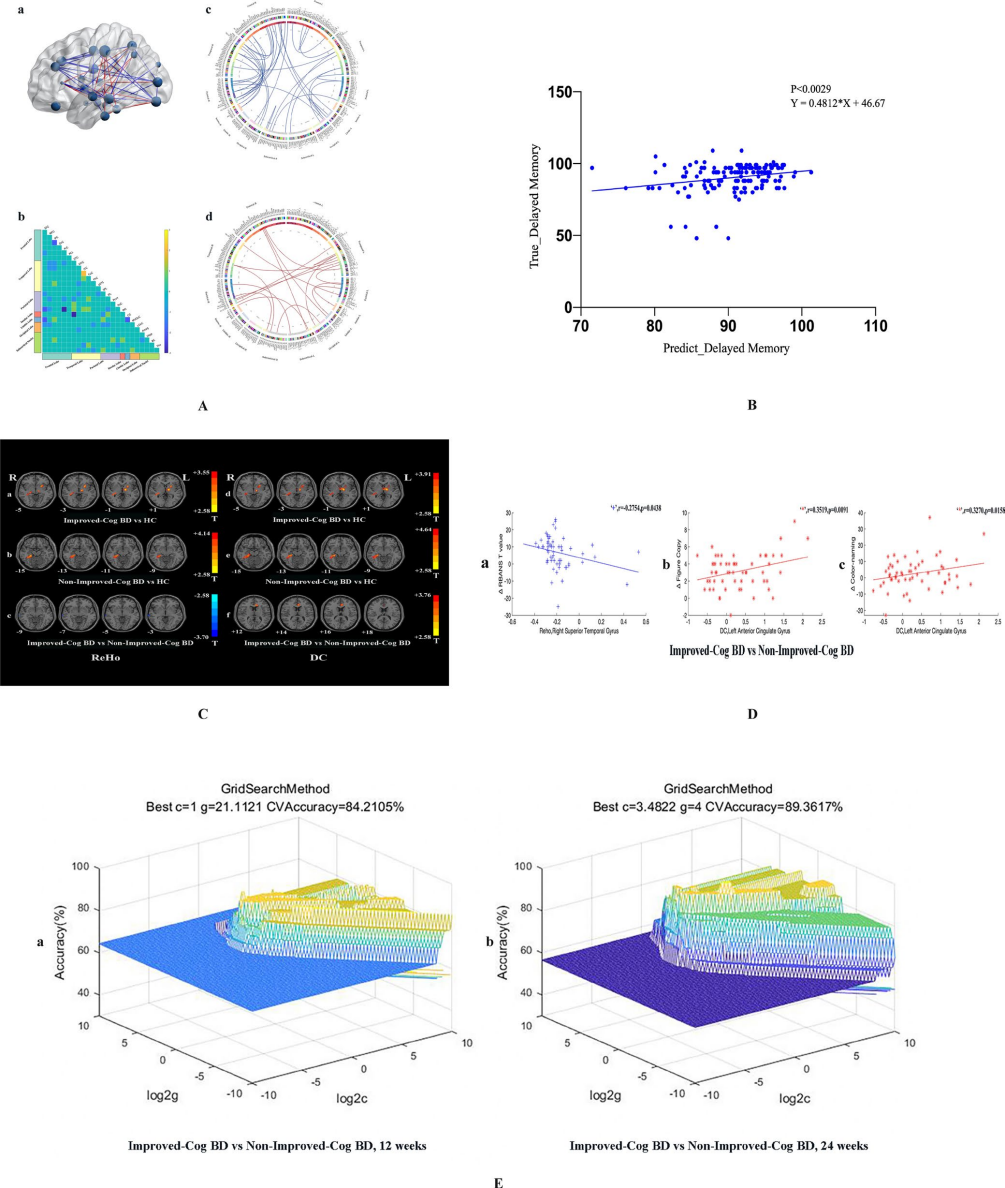
Comparison between Improved‐Cog BD and Non‐Improved‐Cog BD. A: Visualizing selected connectivity features. (a) Glass brain plots: The size of the node represents the number of edges connected to each brain region, and the color of the line represents positive or negative correlation. The positive features (edges) are coded red, and the negative features (edges) are coded blue. (b) Matrix plots: The cells of the matrix plots represent the difference between the total number of negative and positive edges connecting the nodes in different brain regions. (c) and (d) Circle plots: 80 edges (c) negative correlated with the delayed memory scores of the RBANS with a typical significance threshold of *p* = 0.001, and 36 edges (d) positive correlated with the delayed memory scores of the RBANS. B: The CPM model predicted the delayed memory scores of the patients with BD in baseline (*r* = 0.4812, *p* < 0.0029). C: Significant Differences in ReHo and DC across Groups. Brain regions showing significant ReHo (left) and DC (right) differences across Groups. Red denotes higher ReHo/DC values in patients compared to the HC. The color bars indicate the T values of the one‐way analysis of variance (ANOVA). D: Correlations between abnormal ReHo/DC values and clinical variables. Pearson correlation analysis was used to calculate the correlation between abnormal ReHo and DC values and clinical variables in patients with BD and the HCs. Age, gender, education level, and Framewise Displacement (FD) value were used as covariates. In both cases, the correlation analyses were Bonferroni corrected. “+” denotes ReHo values and “*” denotes DC values. Delta means the difference before and after 12 weeks of treatment. E: 3D view of the classified accuracy with the best parameters for identifying Improved‐Cog BD from Non‐Improved‐Cog BD using the ReHo values in the right superior temporal gyrus and the DC values in the left anterior cingulate gyrus. Left: To identify Improved‐Cog BD from Non‐Improved‐Cog BD after 12 weeks of treatment. Right: To identify Improved‐Cog BD from Non‐Improved‐Cog BD after 24 weeks of treatment. BD, the patients with bipolar disorder; CPM, connectome‐based predictive modeling; DC, degree centrality; HC, healthy controls; Improved‐Cog BD, the improved cognitive function patients with BD; Non‐Improved‐Cog BD, the nonimproved cognitive function patients with BD; RBANS, Repeatable Battery for the Assessment of Neuropsychological Status; ReHo, regional homogeneity; SVM, support vector machine.

CPM analysis (Figure 3A) identified 80 edges that were negatively correlated with the delayed memory scores of the RBANS at a significance threshold of *p* = 0.001. In addition, 36 edges were positively correlated with the delayed memory scores of the RBANS. The distribution of all connected edges in each brain region based on the BNA template, where the size of the node represents the number of edges connected to each region, and the color of the line represents a positive or negative correlation. Figure 3A(b) displayed the predefined brain regions based on the BNA template. The cells of the matrices indicated the difference between the total number of negative and positive edges connecting the nodes in different brain regions.

The CPM model based on the negative edges predicted the delayed memory scores of patients with BD. These negative edges were distributed across various brain regions, including the frontal lobe, temporal lobe, parietal lobe, insular lobe, limbic lobe, occipital lobe, and subcortical nuclei. Figure 3B demonstrated the prediction of the delayed memory scores of patients with BD at baseline using the CPM model (*r* = 0.4812, *p* < 0.0029).

### 3.4 Grouping Results Based on Cognitive Function Improvement

Follow‐up assessment at 12 and 24 weeks showed that lower scores of the RBANS T values, immediate memory, visuospatial, and delayed memory scores in patients with BD were found compared with baseline. However, no significant changes in language and attention scores were observed compared with baseline (Table S2). In this study, improvement in cognitive function was defined on the basis of changes in RBANS T values. The reliable change index for the RBANS T value was 9.91, indicating that a change of more than 10 points confirms a real change. The significant cutoff was determined to be 89.73 (Table S3).

At 12‐week follow‐up, 24 patients with BD showed significant improvement in cognitive function, whereas 33 patients with BD did not meet the criteria. Similarly, at 24‐week follow‐up, 25 patients with BD demonstrated clinically significant improvement in cognitive function, whereas 22 patients with BD did not meet the criteria. No significant differences in demographic characteristics or concurrent medication usage were found between the Improved‐Cog BD and Non‐Improved‐Cog BD groups at baseline, 12 weeks, and 24 weeks (Table S4).

In addition, no significant differences in the performance of baseline cognitive function were found between the Improved‐Cog BD and Non‐Improved‐Cog BD groups after 12 weeks of treatment. The differences in demographic and clinical characteristics across groups at baseline are detailed in Table S5.

### 3.5 Differences in ReHo and DC Across Groups

Table 2 and Figure 3C show that the Improved‐Cog BD and Non‐Improved‐Cog BD groups after 12 weeks of treatment exhibited increased ReHo and DC values in the right hippocampus/right parahippocampal gyrus compared with HC. In addition, the Improved‐Cog BD group after 12 weeks of treatment showed increased ReHo values in the left putamen/left thalamus compared with HC. Compared with the Non‐Improved‐Cog BD groups, the Improved‐Cog BD group after 12 weeks of treatment exhibited decreased ReHo values in the right superior temporal gyrus and increased DC values in the left ACC.

### 3.6 Correlations Between Abnormal ReHo/DC Values and Clinical Variables

Significant negative correlations were found between increased ReHo/DC values in the right hippocampus/right parahippocampal gyrus and the scores of immediate memory, language, RBANS total scores, RBANS T value, and delayed memory in patients with BD and HC. Conversely, a positive correlation was found between decreased ReHo and DC values in the left middle frontal gyrus and delayed memory scores (Figure 2c–h). The Improved‐Cog BD and Non‐Improved‐Cog BD groups showed a negative correlation between decreased ReHo values in the right superior temporal gyrus at baseline and the change in RBANS T scores before and after 12 weeks of treatment. In addition, a positive correlation was found between increased DC values at baseline in the left ACC of the Improved‐Cog BD and Non‐Improved‐Cog BD groups after 12 weeks of treatment. The change in figure copy scores and color‐naming scores before and after treatment is shown in Figure 3D.

### 3.7 SVM Results

In Figure 3E, it is shown that 18 out of 24 Improved‐Cog BD exhibited decreased ReHo values in the right superior temporal gyrus at baseline. For Non‐Improved‐Cog BD, the figure was 29 out of 33. Similarly, after 12 weeks of treatment, 17 out of 24 Improved‐Cog BD and 31 out of 33 Non‐Improved‐Cog BD exhibited increased DC values in the left ACC at baseline. The decreased ReHo values in the right superior temporal gyrus and the increased DC values in the left ACC at baseline indicated that a correct classification of 16 out of 24 Improved‐Cog BD and 32 out of 33 Non‐Improved‐Cog BD was achieved after 12 weeks of treatment. It resulted in an optimal sensitivity of 66.77%, an optimal specificity of 96.97%, and an accuracy of 84.21%.

After 24 weeks of follow‐up, the decreased ReHo values in the right superior temporal gyrus and the increased DC values in the left ACC at baseline showed that 22 out of 25 Improved‐Cog BD and 20 out of 22 Non‐Improved‐Cog BD were found, resulting in an optimal sensitivity of 88.00%, an optimal specificity of 90.91%, and an accuracy of 89.36%.

## 4 Discussion

The present study revealed that patients with BD exhibited abnormal brain activities in the frontal‐limbic network compared with HC. This was related to the cognitive function of patients with BD. The CPM model, established by the negative edge associated with delayed memory, predicts the delayed memory score of the RBANS in patients with BD. Compared with the Non‐Improved‐Cog BD group, Improved‐Cog BD after 12 weeks of treatment exhibited abnormal activities in the right superior temporal gyrus and the left ACC at baseline. It is noted that SVM could be used for the treatment of cognitive function in patients with BD.

In this study, some findings needed to be highlighted. First, two indicators, ReHo and DC, were studied to reflect brain function activity. These two indicators showed high consistency in the analysis results (Figure S1). Second, two machine learning methods, CPM and SVM, were used simultaneously. The models constructed using CPM and SVM demonstrated excellent performance in prediction. Furthermore, SVM exhibited excellent predictive ability for additional new samples at 24 weeks of treatment. Finally, for the first time, mathematical methods were used to calculate the criteria for significant clinical improvement in cognitive function among patients with BD. Based on these criteria, participants were grouped to compare the differences in baseline brain function in a direct manner instead of correlational comparisons conducted in previous studies.

The results that cognitive impairment in patients with BD covers most cognitive domains are consistent with most previous studies. Patients with BD showed significantly lower memory (including immediate and delayed memory), visuospatial, language, attention, and processing speed (PS) compared with HC. The overall cognitive function of participants was evaluated by using the total score of RBANS converted into standardized scores. Although classical assessment methods, such as the Digit Symbol Substitution Test, Stroop, and the language fluency of RBANS, were not adopted to valuate PS directly in this study, the PS in cognitive impairment was presented [51]. Although the baseline period of the samples in this study was all young patients who did not receive medication or physical therapy, the impairment still exists. Since only two cognitive tools, RBANS and Stroop, were used to evaluate the cognitive function of participants, some cognitive function may be neglected. Cognitive impairment in BD is a clinical issue that urgently needs to be addressed.

It may be an important way to study cognitive impairment in BD through brain functional activity. In this study, consistent changes in ReHo and DC values in the right hippocampus/right parahippocampal gyrus and the left middle frontal gyrus in patients with BD were reported. It was rarely mentioned in previous research. These findings highlight the frontal‐limbic network in the neurobiological mechanism of patients with BD. The hippocampus/parahippocampal gyrus and middle frontal gyrus play important roles in emotion and cognitive function [52, 53, 54, 55]. In previous studies, increased activities in the hippocampus/parahippocampal gyrus and decreased activities in the mPFC of patients with BD were reported [56, 57, 58, 59]. The results were consistent with our findings. Hyperactivity in the hippocampus/right parahippocampal gyrus may be a compensatory functional enhancement caused by early damage to the hippocampal structure since the volumes of the hippocampus in patients with BD have significantly reduced [60, 61]. From a network perspective, hypoactivity in inhibitory structures such as the middle frontal gyrus may be associated with hyperactivity in the entire frontal‐limbic network, including the hippocampus. These activities may typically be suppressed [62]. This finding may also be related to the sequential order of the evolution of the hippocampus and the mPFC [63]. Moreover, in this study, a negative correlation between increased activities in the hippocampus/parahippocampal gyrus and cognitive function in patients with BD was detected, while a positive correlation between decreased activities in the mPFC and cognitive function was found. This result consolidated our proposed mechanism. An animal study conducted on rhesus monkeys also showed similar findings. It is indicated that the mPFC and hippocampus were involved in cognitive functions despite the different roles they played [64]. Abnormalities in the frontal‐limbic network were associated with emotional and cognitive processing. However, a correlation between abnormal activity in the frontal‐limbic network and clinical symptoms in patients with BD is not yet found. This discrepancy may be attributed to the heterogeneity of BD and its various disease subtypes.

The CPM approach has several strengths including linear operations and a purely data‐driven approach. Linear operations allow fast computation, straightforward interpretation of feature weights, and simple software implementation. Compared with hypothesis‐driven approaches that focus on specific edges, regions, or networks of interest, CPM considers the entire brain and identifies the most relevant features (edges) for predicting outcomes. This comprehensive approach didn't avoid many connections and potentially enhances predictive power [65, 66, 67, 68]. Furthermore, the CPM approach allows for clear interpretation of the predictive networks from the analysis. This runs contrary to multivariate methods [32]. The negative edges that contributed to delayed memory scores included the frontal lobe, temporal lobe, parietal lobe, insular lobe, limbic lobe, occipital lobe, and subcortical nuclei in our study. In addition, the FC of the frontal‐limbic network related to delayed memory was extracted to develop a predictive model. But it fails to predict the cognitive function of patients with BD. In the future, a larger sample size will be involved to utilize CPM for predication. Nevertheless, in our study the CPM approach was adopted to develop a predictive model of cognitive function, indicating that delayed memory scores are obtained by inputting the patient's fMRI information [32]. This approach provides an opportunity for us to rapidly assess the cognitive function of patients with BD in a clinical setting.

After 12 weeks of treatment, the Improved‐Cog BD and Non‐Improved‐Cog BD groups exhibited increased ReHo and DC values in the right hippocampus/right parahippocampal gyrus at baseline compared with the HC. These abnormal activities in the right hippocampus/right parahippocampal gyrus may reflect an inherent characteristic of patients with BD, as this change was also observed in the 92 patients with BD. However, no significant differences in abnormal activities in the right hippocampus/right parahippocampal gyrus were found between the Improved‐Cog BD group and the Non‐Improved‐Cog BD group. Although abnormal activities in the right hippocampus/right parahippocampal gyrus are associated with cognitive function in patients with BD, they hardly predict the treatment efficacy of cognitive function after 12 weeks. After 12 weeks of treatment, the Improved‐Cog BD group exhibited increased ReHo values in the left putamen/left thalamus at baseline compared with the HC. The thalamus, which is part of the limbic system, is closely interconnected with the putamen with regard to structure [69]. Recent research has also indicated the association of the thalamus with cognitive function [70, 71]. Low prefrontal cortex‐thalamic anatomical connectivity has been observed in early‐stage psychotic BD to show compromised thalamic structure in patients with BD [72]. One previous study found the same results that increased ReHo values in the thalamus of patients with BD [73] were discovered. The increased activity in this region at baseline was associated with the treatment response in cognitive function, potentially explaining our results.

The superior temporal gyrus plays a crucial role in the pathway involving the prefrontal cortex and amygdala during social cognition [74, 75]. It is also part of the broader limbic system structure. Studies have found that patients with BD show decreased ReHo values in the superior temporal gyrus. This indicator is important for cognitive processing and affective functions [76, 77]. Similarly, in this study, a negative correlation between decreased ReHo values in the right superior temporal gyrus at baseline and the change values of RBANS T scores was detected before and after treatment in the Improved‐Cog BD group after 12 weeks of treatment. Therefore, hypoactivity in the superior temporal gyrus during the early stages of the illness indicates better treatment efficacy in cognitive function among patients with BD. Anatomically, the ACC is divided into cognitive (dorsal) and emotional (ventral) components [78]. The dorsal part of the ACC is connected to the prefrontal cortex and parietal cortex, while the ventral part is connected to the amygdala and hippocampus [79, 80, 81]. The ACC has been linked to the neurobiology of BD, influencing emotions and cognitive processes [82]. The Improved‐Cog BD group after 12 weeks of treatment showed increased DC values in the left ACC at baseline. The abnormal activities in the ACC, which is connected to the hippocampus with high activities and the middle frontal gyrus with low activities in patients with BD, may be attributed to these connections [83]. As mentioned before, the intrinsic structural and functional connections within the ACC and the frontal‐limbic network can justify our findings. A positive correlation was found between increased DC values in the left ACC and the change values of figure copy scores and color‐naming scores in our study. Another study by Karianne et al. also found a correlation between abnormal activities in the ACC and behavioral scores [84]. In addition, a structural MRI study found that the abnormal structure of the ACC (gray and white matter) in patients with BD correlated with cognitive test performance [85]. The compensatory increase in function for structural abnormalities may explain this finding [86]. Therefore, the aberrant local activities in the right superior temporal gyrus and left ACC may serve as intrinsic neuroimaging biomarkers for predicting the treatment efficacy of cognitive function in patients with BD. This study provides a neuroimaging basis for exploring the neuropathological mechanism of cognitive impairment and identifying potential targets for cognitive impairment treatment in patients with BD.

SVM not only predicted the efficacy of cognitive function treatment in patients with BD after 12 weeks but also provided a better prediction after 24 weeks of treatment. This finding is interesting because the samples at 24 weeks of treatment were considered new for the SVM model, though they are not entirely independent. Therefore, the right superior temporal gyrus and left ACC could be used as neuroimaging biomarkers to predict the treatment efficacy of cognitive function in patients with BD. For individual patients with BD, ReHo and DC values of relevant brain regions were calculated on the basis of their brain fMRI. It is subsequently utilized as indicators to predict potential treatment efficacy of cognitive function. This approach provides an approach for identifying treatment targets related to cognitive impairments in patients with BD and assists the Non‐Improved‐Cog BD group to practice early cognitive function enhancement training.

However, this study has limitations. First, the participants included patients with BD at different disease stages. Although we made a balanced distribution of disease stages across the groups, the analysis results may be influenced. Second, our study lacked a control group of patients who did not receive the same treatment that an excellent control group demands. However, obtaining samples from this group in clinical practice is challenging. Most of the patients and their families seeking medical treatment in hospitals expect treatment. Third, our study lacked longitudinal MRI information that limits our ability to assess changes in brain function over time. Fourth, the relationship between structural changes and cognitive functions in patients with BD was not analyzed. Finally, our machine learning model lacked independent sample validation, which reduces the replicability of the model to a certain extent.

## 5 Conclusion

This study suggests that dysfunction in the frontal‐limbic network may be a significant factor in the neuropathological mechanism of cognitive impairment in patients with BD. Furthermore, the right superior temporal gyrus and the left ACC have been identified as potential neuroimaging biomarkers. These biomarkers could be used to predict the efficacy of treatments targeting cognitive function, offering a promising avenue to address cognitive impairment in patients with BD.

## Author Contributions

Sujuan Li: Data curation, formal analysis, methodology, and writing – original draft. Yangpan Ou: Methodology, software, visualization, and writing – review and editing. Haiping Liu, Qianyu Dong, Yan Qiu, Ziwei Teng, Hui Tang, Hui Xiang, Guowei Wu, and Lutao Jiang: Methodology and supervision. Jindong Chen: Conceptualization and funding acquisition. Bolun Wang: Methodology. Haishan Wu: Conceptualization, funding acquisition, methodology, and writing – review and editing.

## Funding

This work was supported by National Natural Science Foundation of China (82271571), the China Postdoctoral Science Foundation (2025M772098), and Joint Funding Project of Hunan Provincial Natural Science Foundation and Hunan Xiangya Boai Rehabilitation Hospital (2024JJ9109, 2025JJ90282).

## Ethics Statement

All participants in this study provided a written informed consent. The study was approved by the ethics committee of the Second Xiangya Hospital of Central South University in Changsha, China (ID: 2019 (K106)).

## Conflicts of Interest

The authors declare no conflicts of interest.

## Supporting information


**Appendix S1:** cns70730‐sup‐0001‐AppendixS1.docx.

## Data Availability

The data that support the findings of this study are available from the corresponding author upon reasonable request.
