# Supplementary material for: Differences in the Efficacy of Cognitive Function Treatment Related to Functional Patterns in the Frontal‐Limbic Network in Patients With Bipolar Disorder
Source: CNS Neurosci Ther. 2026 Jan 5;32(1):e70730. doi: 10.1002/cns.70730 (PMC12766901; doi:10.1002/cns.70730)
Supplement: Supplementary file 1 — Appendix S1: cns70730‐sup‐0001‐AppendixS1.docx. [file CNS-32-e70730-s001.docx]

**Differences in the Efficacy of Cognitive Function Treatment Related to Functional Patterns in the Frontal-limbic Network in Patients with Bipolar Disorder**

Sujuan Li^a#^, Yangpan Ou^a#^, Haiping Liu^b^, Qianyu Dong^a^, Yan Qiu^c^, Ziwei Teng^d^, Hui Tang^a^, Hui Xiang^a^, Jindong Chen^a^, Bolun Wang^e^, Lutao Jiang^f^, Haishan Wu ^af^*

1. Department of Psychiatry, National Clinical Research Center for Mental Disorders, and National Center for Mental Disorders, The Second Xiangya Hospital of Central South University, Changsha 410011, Hunan, China.
2. Oingdao Mental Health Center, Qingdao 266034, Shandong, China
3. Xiamen Xianyue Hospital, Xianyue Hospital Affliated with Xiamen Medical College,"uiian Psvchiatric Center, Fuiian Clinical Research Center for Mental Disorders, &iamer361012, Fujian, China.
4. Department of Psychiatry, Clinical Research Center for Depressive Disorder in Hunan Province, The Second People's Hospital of Hunan Province (Brain Hospital of Hunan Province), Changsha 410007, China.
5. Department of Radiology, The Second Xiangya Hospital of Central South University, Clinical Research Center for Medical Imaging in Hunan Province, Scientific Research Program of Hunan Provincial Health Commission, Changsha 410011, Hunan, China
6. The Third People's Hospital of Tongren, Tongren 554300, Guizhou, China

***Corresponding author:**

Haishan Wu, MD, PhD, The Second Xiangya Hospital of Central South University, Changsha, 410011, China. Email: [wuhaishan@csu.edu.cn](mailto:wuhaishan@csu.edu.cn)

#These authors have contributed equally to this work and share co-first authorship.

**SUPPLEMENTARY MATERAIL**

**Methods**

**2.1 Healthy Controls**

Inclusion criteria for the healthy control group were as follows: (1) individuals aged between 18 and 50 years old; (2) Chinese Han nationality; (3) no previous diagnosis or family history of neuropsychiatric disorders; (4) never treated with psychiatric pharmacotherapy; and (5) absence of severe physical illnesses such as heart disease or metabolic syndrome.

**2.2** **Clinical and Cognitive Assessment**

We utilized the RBANS to assess neuropsychological functioning. The RBANS consists of 12 subtests across five domains: immediate memory (list learning, story memory), visuospatial (figure copy and line orientation), language (picture naming and semantic fluency), attention (digit span and coding tasks), and delayed memory (list recall, list recognition, story recall, and figure recall) (1). To standardize the scores, we converted the raw scores (total score and scores for each domain) into T scores using established norms. Additionally, the Stroop color-word test, which evaluates working memory capacity, conflict monitoring, and speed of visual search, was administered across three domains: word reading, color naming, and color words (2). The Chinese version of the RBANS and Stroop color-word test underwent reliability and validity evaluation (3-5).

**2.4 Image Acquisition and Imaging Data Processing**

Resting-state fMRI images were acquired using a gradient-echo echo-planar imaging sequence with the following parameters: repetition time/echo time = 2,000 ms/30 ms, 33 slices, 64 × 64 matrices, 90° flip angle, 22 cm field of view, 4 mm slice thickness, no slice gap, and 240 volumes (480 s). The acquired images underwent preprocessing using DPABI software in MATLAB (R2018b) (6). Preprocessing steps included slice timing correction, head motion correction, and exclusion of participants with maximal translation exceeding 2 mm or maximal rotation exceeding 2°. Several covariates were removed, including Friston-24 head motion parameters, signal from a ventricular region of interest, and signal from a white matter-centered region. Global signal removal was not performed due to the ongoing controversy in the field of resting-state fMRI. The individual functional images underwent spatial normalization, aligning them with the Montreal Neurological Institute (MNI) space. This was achieved by applying the transformation matrix obtained from registering the T1 image (co-registered with functional images) to the MNI template using unified segmentation The resulting normalized images had a voxel size of 3 mm x 3 mm x 3 mm. Finally, the images were bandpass-filtered (0.01–0.1 Hz) and linearly detrended.

**2.6 CPM Analysis**

**2.6.1 Feature selection, Summarization, and Model Building using CPM**

Step 1: Load the connectivity matrices and behavioral data (RBANS scores) into MATLAB for analysis. The connectivity matrices for all subjects should be contained in a 3D matrix with dimensions of M × M × N, where M represents the number of nodes used in the connectivity analysis, and N represents the number of subjects. The behavioral variable should be stored in an N × 1 array.

Step 2: Split the data into training and testing sets for cross-validation. In this study, a leave-one-subject-out cross-validation method was employed. In each iteration, one subject was excluded from the training data, and the predictive model was built using the remaining N-1 subjects. This process was repeated with a different subject left out each time.

Step 3: Utilize linear regression (specifically, Pearson's correlation) to examine the connectivity matrices for each edge and their relationship to the behavioral measure. This analysis is conducted across all subjects in the training set.

Step 4: Select the most relevant edges for the predictive model based on the correlation analysis between each edge in the connectivity matrices and the behavioral measure. Edge selection is typically based on the significance of the association between the edge and behavior, using a significance threshold (P = 0.001) to determine the most relevant edges.

Step 5: Summarize the selected edges for each individual in the training set to obtain a single value per subject for the positive and negative edge sets separately. This can be achieved by masking the individual connectivity matrices with the chosen positive and negative edges and summing the corresponding edge-strength values (similar to computing the dot product between an individual's connectivity matrices and the binary feature masks generated earlier).

Step 6: Fit the model by assuming a linear relationship between the behavioral variable (dependent variable) and the single-subject summary value (independent variable). This process can be carried out separately for both the positive and negative edge sets.

Step 7: To predict performance on novel subjects, estimate a predictive model and calculate a summary value for each subject in the testing set. Input the summary value into the model to obtain the predicted behavioral measure for that subject. Predictions are made separately for each testing subject in both the positive and negative edge sets, specifically for the Delayed Memory scores.

Step 8: Evaluate the accuracy of the predictive model by comparing the predicted behavioral values for all subjects in the testing set with the true observed values. Permutation testing is used to assess the statistical significance of the correlation between the predicted and observed values in this study.

**2.6.2 Assessment of Prediction Significance**

Perform 5000 iterations by shuffling the data labels, calculating the correlation coefficient, and determining the proportion of sampled permutations that are greater than or equal to the true prediction correlation. This provides the P value for the permutation test.

**2.6.3 Generation of Circos Plots**

Circos plots based on FC (functional connectivity) matrices were generated using Strawberry Perl.

**SUPPLEMENTARY TABLES**

***Supplementary Table S1*** Demographic and Clinical Characteristics Data of subtype.^a^

| Variable | Depressed (n=63) | | Manic (n=7) | | Mixed (n=22) | | c2 | *p* |
| --- | --- | --- | --- | --- | --- | --- | --- | --- |
|  | N | % | N | % | N | % |  |  |
| **Demographic Characteristic** |  |  |  |  |  |  |  |  |
| Gender |  |  |  |  |  |  | 0.971 | 0.615 |
| Male | 16 | 25.4 | 3 | 42.9 | 6 | 27.3 |  |  |
| Female | 47 | 74.6 | 4 | 57.1 | 16 | 72.7 |  |  |
|  | Mean | SD | Mean | SD | Mean | SD | F | *p* |
| Age (y) | 20.59 | 3.43 | 19.86 | 4.30 | 20.68 | 5.28 | 0.120 | 0.887 |
| Education(y) | 14.08 | 1.95 | 14.57 | 2.15 | 13.14 | 2.21 | 2.184 | 0.119 |
| Disease Duration(y) | 2.90 | 1.52 | 2.51 | 1.63 | 3.00 | 1.51 | 0.270 | 0.764 |
| Framewise Displacement | 0.08 | 0.03 | 0.09 | 0.04 | 0.08 | 0.03 | 0.521 | 0.596 |
| **Clinical Characteristic** |  |  |  |  |  |  |  |  |
| HAMD-17 | 24.02 | 6.17 | 9.00 | 6.54 | 23.45 | 6.53 | 18.184 | <0.001 |
| YMRS | 6.33 | 3.56 | 20.57 | 5.35 | 16.64 | 5.10 | 76.897 | <0.001 |
| HCL-32 | 18.51 | 6.29 | 20.86 | 3.98 | 19.91 | 5.42 | 0.809 | 0.449 |
| **RBANS T Value** | 85.46 | 10.03 | 81.86 | 14.10 | 82.82 | 9.96 | 0.790 | 0.457 |
| **RBANS Total Score** | 446.16 | 41.03 | 430.71 | 54.79 | 434.36 | 41.70 | 0.913 | 0.405 |
| **Immediate Memory** | 84.13 | 14.25 | 82.43 | 15.09 | 83.36 | 13.60 | 0.060 | 0.942 |
| List Learning | 28.97 | 5.51 | 28.43 | 4.72 | 29.18 | 4.43 | 0.056 | 0.946 |
| Story Memory | 14.19 | 3.89 | 12.57 | 5.41 | 13.41 | 3.53 | 0.742 | 0.479 |
| **Visuospatial** | 72.06 | 8.10 | 66.00 | 5.54 | 68.45 | 6.42 | 3.328 | 0.040 |
| Figure Copy^§^ | 14.76 | 1.20 | 12.57 | 2.44 | 14.45 | 1.26 | 7.144 | 0.028 |
| Line Orientation | 16.38 | 2.84 | 14.43 | 2.37 | 15.36 | 3.19 | 2.102 | 0.127 |
| **Language** | 89.35 | 15.54 | 87.29 | 17.41 | 84.95 | 17.51 | 0.614 | 0.543 |
| Picture Naming | 8.97 | 0.88 | 9.29 | 0.76 | 8.68 | 0.89 | 1.521 | 0.224 |
| Semantic Fluency | 19.76 | 4.43 | 17.71 | 6.82 | 19.27 | 3.69 | 0.693 | 0.503 |
| **Attention** | 112.44 | 11.93 | 109.57 | 18.52 | 110.91 | 11.63 | 0.253 | 0.777 |
| Digit Span | 15.00 | 1.33 | 14.86 | 1.57 | 15.09 | 1.15 | 0.091 | 0.913 |
| Coding Tasks | 58.02 | 9.17 | 54.68 | 14.63 | 56.27 | 7.78 | 0.555 | 0.576 |
| **Delayed Memory** | 88.17 | 10.70 | 85.43 | 15.04 | 86.68 | 8.58 | 0.322 | 0.726 |
| List Recall | 7.06 | 1.94 | 7.57 | 2.76 | 7.27 | 1.42 | 0.283 | 0.754 |
| List Recognition | 19.75 | 0.70 | 19.57 | 0.79 | 19.59 | 1.10 | 0.384 | 0.682 |
| Story Recall | 7.87 | 2.37 | 7.71 | 2.14 | 8.00 | 1.75 | 0.051 | 0.951 |
| Figure Recall | 12.81 | 2.81 | 11.57 | 3.46 | 11.68 | 2.77 | 1.639 | 0.200 |
| **Stroop Total Score** | 211.33 | 32.22 | 208.29 | 43.70 | 208.38 | 38.74 | 0.070 | 0.932 |
| Word-reading | 99.23 | 16.21 | 93.71 | 20.65 | 94.10 | 18.41 | 0.900 | 0.410 |
| Color-naming | 70.28 | 12.14 | 74.00 | 15.62 | 74.10 | 15.04 | 0.801 | 0.452 |
| Color-word | 41.82 | 9.75 | 40.57 | 13.44 | 40.19 | 10.36 | 0.221 | 0.802 |

Test statistics: chi-square test for categorical variables, ANOVA (equal variances assumed) and Kruskal-Wallis one-way ANOVA(equal variances not assumed, §) for continuous variables.

BD = The patients with bipolar disorder, HC = Healthy controls.

***Supplementary Table S2.*** Comparison of RBANS Scores at Different Time Periods^a^

| Variable | Baseline  (n=92) | | Follow-up | | | | Baseline vs 12 weeks | | Baseline vs 24 weeks | |
| --- | --- | --- | --- | --- | --- | --- | --- | --- | --- | --- |
|  |  |  | 12 weeks  (n=57) | | 24 weeks  (n=47) | |  |  |  |  |
|  | Mean | SD | Mean | SD | Mean | SD | t | *p* | t | *p* |
| RBANS T value | 84.55 | 10.32 | 90.91 | 14.00 | 93.88 | 13.55 | -3.182 | 0.002 | -4.669 | <0.001 |
| Immediate Memory | 83.82 | 14.01 | 93.18 | 16.92 | 99.67 | 15.86 | -3.657 | <0.001 | -6.111 | <0.001 |
| Visuospatial | 70.74 | 7.77 | 85.02 | 13.90 | 83.02 | 12.45 | -7.097 | <0.001 | -6.285 | <0.001 |
| Language | 88.14 | 16.09 | 85.04 | 15.19 | 87.20 | 15.88 | 1.170 | 0.244 | 0.331 | 0.741 |
| Attention | 111.86 | 12.32 | 109.70 | 12.55 | 112.55 | 15.28 | 1.031 | 0.304 | -0.292 | 0.771 |
| Delayed Memory | 95.00 | 6.03 | 93.19 | 13.47 | 95.49 | 10.61 | -2.824 | 0.005 | -4.222 | <0.001 |

***Supplementary Table*** ***S3*** The Reliable Change Index of RBANS.

| Variable | Mean_clin_ | SD_clin_ | Mean_norm_ | SD_norm_ | α | SE_diff_ | RC_index_ | CS_cut-off_ |
| --- | --- | --- | --- | --- | --- | --- | --- | --- |
| RBANS T value | 84.55 | 10.32 | 94.38 | 9.27 | 0.88 | 5.05 | 9.91 | 89.73 |
| Immediate Memory | 83.82 | 14.01 | 98.04 | 13.90 | 0.86 | 7.41 | 14.53 | 90.96 |
| Visuospatial | 70.74 | 7.77 | 75.05 | 8.92 | 0.68 | 6.22 | 12.19 | 72.75 |
| Language | 88.14 | 16.09 | 95.76 | 12.80 | 0.67 | 13.07 | 25.61 | 92.39 |
| Attention | 111.86 | 12.32 | 117.89 | 11.40 | 0.85 | 6.75 | 13.22 | 114.99 |
| Delayed Memory | 87.61 | 10.52 | 95.00 | 6.03 | 0.80 | 6.66 | 13.05 | 92.31 |

$$SE_{\text{diff}}=SD_{1}\times\sqrt{2}\times\sqrt{1-\alpha}$$

$$RC_{\text{index}}=1.96\times SE_{\text{diff}}$$

$$CS_{\text{cut}-off}=\frac{(Mean_{clin}\times SD_{norm})+(Mean_{norm}\times SD_{clin})}{SD_{norm}+SD_{clin}}$$

SD_1_ is the standard deviation of the scale at baseline and Cronbach’s α is the reliability of the scale.“clin” and “norm” stand for clinical and normative.

The reliable change index of RBANS T value was 9.91 (rounded-off to 10), indicating that a >10-point change is needed to state with 95% confidence that a real change has occurred in a patient and the clinically significant cutoff was 89.73 (rounded-off to 90).

***Supplementary Table S4.***  Demographic and Clinical Characteristic Data Between Groups at Baseline, 12 Weeks, and 24 Weeks.

| Variable | Improved-Cog BD | | | | | | Non-Improved-Cog BD | | | | | | A_0_ vs B_0_ | | A_1_ vs B_1_ | | A_2_ vs B_2_ | |
| --- | --- | --- | --- | --- | --- | --- | --- | --- | --- | --- | --- | --- | --- | --- | --- | --- | --- | --- |
|  | Baseline  (A_0_, n=24) | | 12 weeks  (A_1_, n=24) | | 24 weeks  (A_2_, n=25) | | Baseline  (B_0_, n=33) | | 12 weeks  (B_1_, n=33) | | 24 weeks  (B_2_, n=22) | |  |  |  |  |  |  |
|  | N | % | N | % | N | % | N | % | N | % | N | % | c2 | *p* | c2 | *p* | c2 | *p* |
| **Demographic Characteristic** |  |  |  |  |  |  |  |  |  |  |  |  |  |  |  |  |  |  |
| Gender |  |  |  |  |  |  |  |  |  |  |  |  | 0.244 | 0.771 | 0.244 | 0.771 | 0.844 | 0.358 |
| Male | 8 | 33.3 | 8 | 33.3 | 10 | 40.0 | 9 | 27.3 | 9 | 27.3 | 6 | 27.3 |  |  |  |  |  |  |
| Female | 16 | 66.7 | 16 | 66.7 | 15 | 60.0 | 24 | 72.7 | 24 | 72.7 | 16 | 72.7 |  |  |  |  |  |  |
| **Subtypes Composition** |  |  |  |  |  |  |  |  |  |  |  |  | 1.217 | 0.544 | 1.217 | 0.544 | 1.636 | 0.441 |
| Depressed | 13 | 54.2 | 13 | 54.2 | 12 | 48.0 | 21 | 63.6 | 21 | 63.6 | 14 | 63.6 |  |  |  |  |  |  |
| Manic | 2 | 8.3 | 2 | 8.3 | 3 | 12.0 | 4 | 12.1 | 4 | 12.1 | 3 | 13.6 |  |  |  |  |  |  |
| Mixed | 9 | 37.5 | 9 | 37.5 | 10 | 40.0 | 8 | 24.2 | 8 | 24.2 | 5 | 22.7 |  |  |  |  |  |  |
| **Concomitant Medication** |  |  |  |  |  |  |  |  |  |  |  |  |  |  |  |  |  |  |
| Typical antipsychotics(yes/no) | - | - | 0 | 0 | 0 | 0 | - | - | 0 | 0 | 0 | 0 | - | - | - | - | - | - |
| Atypical antipsychotics(yes/no) | - | - | 23 | 95.8 | 24 | 96.0 | - | - | 31 | 93.9 | 21 | 95.5 | - | - | 0.100 | 0.752 | 0.009 | 0.926 |
| Mood stabilizers(yes/no) | - | - | 24 | 100.0 | 25 | 100.0 | - | - | 32 | 97.0 | 22 | 100 | - | - | 0.740 | 0.390 | - | - |
| Antidepressants(yes/no) | - | - | 0 | 0 | 0 | 0 | - | - | 0 | 0 | 0 | 0 | - | - | - | - | - | - |
| Anticholinergica(yes/no) | - | - | 1 | 4.2 | 2 | 8.0 | - | - | 2 | 6.1 | 0 | 0 | - | - | 0.053 | 0.818 | 1.838 | 0.175 |
|  | Mean | SD | Mean | SD | Mean | SD | Mean | SD | Mean | SD | Mean | SD | t | *p* | t | *p* | t | *p* |
| **Demographic Characteristic** |  |  |  |  |  |  |  |  |  |  |  |  |  |  |  |  |  |  |
| Age (y) | 21.63 | 4.62 | 21.63 | 4.62 | 21.56 | 5.19 | 19.94 | 3.98 | 19.94 | 3.98 | 20.23 | 4.32 | 1.428 | 0.159 | 1.428 | 0.159 | 0.946 | 0.348 |
| Education(y) | 14.25 | 1.94 | 14.25 | 1.94 | 14.20 | 1.94 | 13.61 | 1.85 | 13.61 | 1.85 | 13.68 | 2.23 | 1.923 | 0.060 | 1.923 | 0.060 | 0.852 | 0.399 |
| Disease Duration(y) | 3.18 | 1.46 | 3.18 | 1.46 | 3.46 | 1.41 | 2.84 | 1.67 | 2.84 | 1.67 | 2.61 | 1.58 | 0.779 | 0.439 | 0.779 | 0.439 | 1.943 | 0.058 |
| Framewise Displacement | 0.08 | 0.04 | 0.08 | 0.04 | 0.08 | 0.04 | 0.08 | 0.03 | 0.08 | 0.03 | 0.08 | 0.03 | -0.195 | 0.846 | -0.195 | 0.846 | -0.115 | 0.909 |
| **Psychopathology** |  |  |  |  |  |  |  |  |  |  |  |  |  |  |  |  |  |  |
| HAMD-17 | 22.29 | 6.76 | 8.96 | 5.55 | 8.36 | 7.80 | 21.88 | 8.22 | 10.79 | 8.17 | 8.07 | 6.13 | 0.210 | 0.841 | -0.948 | 0.347 | 0.108 | 0.915 |
| YMRS | 10.54 | 6.78 | 3.75 | 4.12 | 3.86 | 4.15 | 10.61 | 6.93 | 6.00 | 6.57 | 4.15 | 4.38 | -0.035 | 0.972 | -1.447 | 0.145 | -0.181 | 0.858 |
| RBANS T value | 86.54 | 10.87 | 100.83 | 13.22 | 102.08 | 11.73 | 82.67 | 10.40 | 83.70 | 9.48 | 85.27 | 9.95 | 1.363 | 0.179 | 5.705 | <0.001 | 5.258 | <0.001 |
| RBANS Total Score | 449.88 | 43.53 | 508.46 | 45.33 | 508.40 | 36.95 | 434.27 | 43.13 | 438.70 | 39.05 | 445.73 | 40.87 | 1.343 | 0.185 | 5.753 | <0.001 | 5.521 | <0.001 |
| Immediate Memory | 84.83 | 14.48 | 100.96 | 18.58 | 106.40 | 15.27 | 83.21 | 14.14 | 13.21 | 87.52 | 91.50 | 13.20 | 0.423 | 0.674 | 3.031 | 0.004 | 3.554 | 0.001 |
| Visuospatial | 72.63 | 7.01 | 91.67 | 15.24 | 89.40 | 12.84 | 69.94 | 8.36 | 80.18 | 10.68 | 76.64 | 8.00 | 1.280 | 0.206 | 3.348 | 0.001 | 4.141 | <0.001 |
| Language | 90.88 | 16.90 | 93.79 | 9.87 | 94.44 | 13.71 | 84.36 | 14.98 | 78.67 | 15.30 | 81.36 | 13.91 | 1.535 | 0.131 | 4.238 | <0.001 | 3.242 | 0.002 |
| Attention | 114.75 | 11.39 | 118.00 | 10.35 | 118.20 | 12.27 | 110.06 | 11.28 | 103.67 | 10.46 | 105.27 | 15.81 | 1.544 | 0.128 | 5.129 | <0.001 | 3.151 | 0.003 |
| Delayed Memory | 86.79 | 8.40 | 99.04 | 13.11 | 99.96 | 7.62 | 86.70 | 13.38 | 88.94 | 12.23 | 90.96 | 11.84 | 0.031 | 0.976 | 2.987 | 0.004 | 3.138 | 0.003 |
| Stroop Total Score | 221.63 | 35.20 | 227.08 | 31.28 | 232.60 | 36.40 | 204.27 | 27.41 | 195.24 | 29.67 | 189.14 | 27.23 | 2.012 | 0.051 | 3.911 | <0.001 | 4.583 | <0.001 |

Improved-Cog BD = The Improved cognitive function patients with bipolar disorder, Non-Improved-Cog BD = The nonimproved cognitive function patients with bipolar disorder.

Test statistics: χ2 test for categorical variables and Student t-test for continuous variables.

***Supplementary Table S5***. Demographic and Clinical Characteristics Data across Groups.

| Variable | Improved-Cog BD (n=24) | | Non-Improved-Cog BD (n=33) | | HC (n=55) | | ANOVA | | Bonferroni | | |
| --- | --- | --- | --- | --- | --- | --- | --- | --- | --- | --- | --- |
|  | N | % | N | % | N | % | χ^2^ | *p* | Improved-Cog BD *vs* HC | Non-Improved-Cog BD *vs* HC | Improved-Cog BD *vs* Non-Improved-Cog BD |
| Gender |  |  |  |  |  |  |  |  |  |  |  |
| Male | 8 | 33.3 | 9 | 27.3 | 20 | 36.4 |  |  |  |  |  |
| Female | 16 | 66.7 | 24 | 72.7 | 35 | 63.6 |  |  |  |  |  |
|  | Mean | SD | Mean | SD | Mean | SD | F | *p* |  |  |  |
| **Demographic Characteristic** |  |  |  |  |  |  |  |  |  |  |  |
| Age (y) | 21.63 | 4.62 | 19.94 | 3.98 | 20.60 | 1.62 | 1.786 | 0.172 | 0.632 | 1.000 | 0.185 |
| Education(y) | 14.25 | 1.94 | 13.61 | 1.85 | 14.32 | 1.04 | 2.393 | 0.096 | 1.000 | 0.112 | 0.348 |
| Disease Duration(y) | 3.18 | 1.46 | 2.84 | 1.67 | - |  | 0.607 | 0.439 | - | - | - |
| **Clinical Characteristic** |  |  |  |  |  |  |  |  |  |  |  |
| HAMD-17 | 22.29 | 6.76 | 21.88 | 8.22 | - |  | 0.041 | 0.841 | - | - | - |
| YMRS | 10.54 | 6.78 | 10.61 | 6.93 | - |  | 0.001 | 0.972 | - | - | - |
| HCL-32 | 19.96 | 5.36 | 19.30 | 5.58 | - |  | 0.198 | 0.658 | - | - | - |
| **RBANS T value** | 86.54 | 10.87 | 82.67 | 10.40 | 94.38 | 9.27 | 15.392 | <0.001 | 0.005 | <0.001 | 0.450 |
| **RBANS Total Score** | 449.88 | 43.53 | 434.27 | 43.13 | 481.75 | 34.73 | 16.269 | <0.001 | 0.004 | <0.001 | 0.425 |
| **Immediate Memory** | 84.83 | 14.48 | 83.21 | 14.14 | 98.04 | 13.90 | 14.188 | <0.001 | 0.001 | <0.001 | 1.000 |
| List Learning | 29.13 | 5.26 | 28.70 | 6.02 | 32.56 | 3.78 | 8.123 | 0.001 | 0.014 | 0.001 | 1.000 |
| Story Memory | 14.13 | 3.63 | 13.67 | 4.31 | 17.55 | 3.17 | 14.486 | <0.001 | 0.001 | <0.001 | 1.000 |
| **Visuospatial** | 72.63 | 7.01 | 69.94 | 8.36 | 75.06 | 8.92 | 3.874 | 0.024 | 0.716 | 0.020 | 0.705 |
| Figure Copy | 14.33 | 1.27 | 14.39 | 1.77 | 16.02 | 1.13 | 20.150 | <0.001 | <0.001 | <0.001 | 1.000 |
| Line Orientation | 16.75 | 3.05 | 15.67 | 3.06 | 16.42 | 2.92 | 1.048 | 0.354 | 1.000 | 0.769 | 0.539 |
| **Language** | 90.88 | 16.90 | 84.36 | 14.98 | 95.76 | 12.80 | 6.481 | 0.002 | 0.504 | 0.001 | 0.284 |
| Picture Naming | 8.96 | 0.91 | 8.82 | 0.95 | 9.09 | 0.80 | 1.026 | 0.362 | 1.000 | 0.472 | 1.000 |
| Semantic Fluency | 20.83 | 4.57 | 18.30 | 3.63 | 21.47 | 4.30 | 6.107 | 0.003 | 1.000 | 0.002 | 0.078 |
| **Attention** | 114.75 | 11.39 | 110.06 | 11.28 | 117.89 | 11.40 | 4.902 | 0.009 | 0.783 | 0.007 | 0.380 |
| Digit Span | 60.08 | 7.41 | 56.33 | 8.57 | 61.98 | 9.06 | 4.468 | 0.014 | 1.000 | 0.010 | 0.319 |
| Coding Tasks | 15.13 | 1.08 | 14.79 | 1.56 | 15.22 | 1.10 | 1.256 | 0.289 | 1.000 | 0.360 | 0.948 |
| **Delayed Memory** | 86.79 | 8.40 | 86.70 | 13.38 | 95.00 | 6.03 | 11.181 | <0.001 | 0.001 | <0.001 | 1.000 |
| List Recall | 7.33 | 1.90 | 7.03 | 2.16 | 8.11 | 1.74 | 3.664 | 0.029 | 0.297 | 0.034 | 1.000 |
| List Recognition | 19.71 | 0.69 | 19.67 | 0.82 | 19.84 | 0.42 | 0.876 | 0.419 | 1.000 | 0.648 | 1.000 |
| Story Recall | 7.38 | 2.24 | 8.15 | 2.35 | 9.69 | 1.75 | 12.610 | <0.001 | <0.001 | 0.003 | 0.482 |
| Figure Recall | 12.25 | 2.42 | 12.27 | 2.92 | 14.31 | 1.55 | 11.897 | <0.001 | 0.001 | <0.001 | 1.000 |
| **Stroop Total Score** | 221.63 | 35.20 | 204.27 | 27.41 | 220.20 | 32.27 | 6.789 | 0.002 | 0.960 | 0.001 | 0.112 |
| Word-reading | 101.75 | 18.42 | 95.06 | 15.89 | 105.40 | 13.16 | 4.763 | 0.010 | 0.987 | 0.008 | 0.312 |
| Color-naming | 75.92 | 13.19 | 70.03 | 10.86 | 77.16 | 13.61 | 3.336 | 0.039 | 1.000 | 0.038 | 0.266 |
| Color-word | 43.96 | 10.54 | 39.18 | 7.91 | 46.56 | 10.82 | 5.636 | 0.005 | 0.866 | 0.003 | 0.232 |

Test statistics: χ2 test for categorical variables and ANOVA for continuous variables.

**SUPPLEMENTARY FIGURES**

***Supplementary Figure S1.*** The consistency between ReHo and DC values in the comparisons between different groups.

**
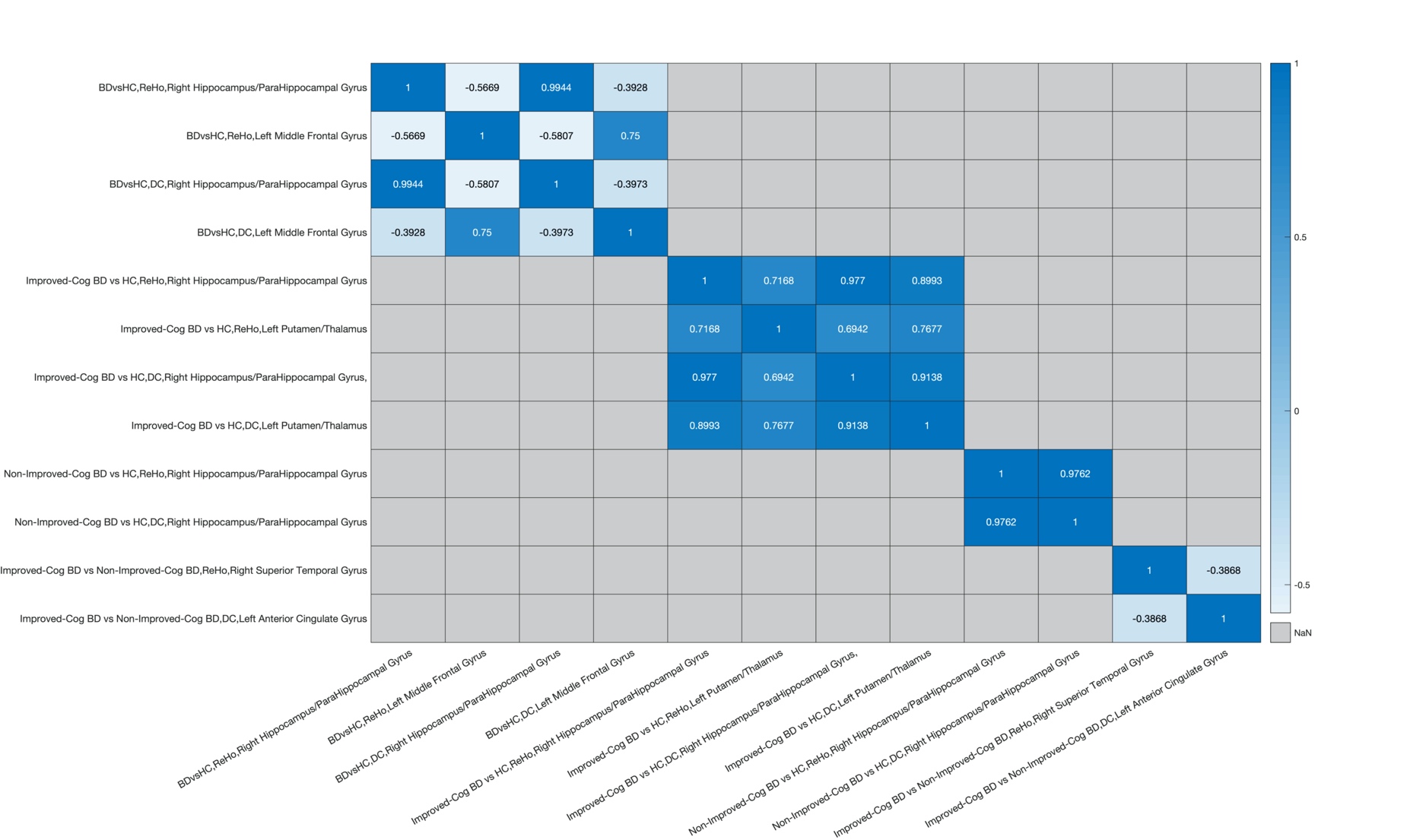
**

Legends: The blue boxes represent the Pearson correlation coefficients between ReHo and DC values in inter-group comparisons of different brain regions. The gray boxes represent invalid values. ReHo = Regional homogeneity, DC = Degree centrality, Improved-Cog BD = The Improved cognitive function patients with bipolar disorder, Non-Improved-Cog BD = The nonimproved cognitive function patients with bipolar disorder.

**References**

1. Randolph C, Tierney MC, Mohr E, Chase TN. The Repeatable Battery for the Assessment of Neuropsychological Status (RBANS): preliminary clinical validity. Journal of Clinical and Experimental Neuropsychology (Neuropsychology, Development and Cognition: Section A). 1998;20(3):310-9.

2. Periáñez JA, Lubrini G, García-Gutiérrez A, Ríos-Lago M. Construct Validity of the Stroop Color-Word Test: Influence of Speed of Visual Search, Verbal Fluency, Working Memory, Cognitive Flexibility, and Conflict Monitoring. Arch Clin Neuropsychol. 2021;36(1):99-111.

3. Phillips R, Cheung YB, Collinson SL, Lim ML, Ling A, Feng L, et al. The Equivalence and Difference between the English and Chinese Language Versions of the Repeatable Battery for the Assessment of Neuropsychological Status. Clin Neuropsychol. 2015;29 Suppl 1:1-18.

4. Zhong N, Jiang H, Wu J, Chen H, Lin S, Zhao Y, et al. Reliability and validity of the CogState battery Chinese language version in schizophrenia. PLoS One. 2013;8(9):e74258.

5. Li X, Shen M, Jin Y, Jia S, Zhou Z, Han Z, et al. Validity and Reliability of the New Chinese Version of the Frontal Assessment Battery-Phonemic. J Alzheimers Dis. 2021;80(1):371-81.

6. Chao-Gan Y, Yu-Feng Z. DPARSF: A MATLAB Toolbox for "Pipeline" Data Analysis of Resting-State fMRI. Front Syst Neurosci. 2010;4:13.
